# Address of the Publishing Committee, to Their Professional Brethren throughout the United States of America

**Published:** 1839-06

**Authors:** 


### THE AMERICAN JOURNAL

OF

### DENTAL SCIENCE.

---

### ADDRESS

### OF THE PUBLISHING COMMITTEE, TO THEIR PROFESSIONAL BRETHREN THROUGHOUT THE UNITED STATES OF AMERICA.

GENTLEMEN :—

The period has at length arrived, when the profession to which we belong assumes a commanding position in the public eye, and challenges successful rivalship with the other useful and profitable avocations of men. This fact is not less auspicious to society at large, than to the professors of Dental Science, and the practitioners of the Dental Art.

Even the Members of the Medical Profession, so proverbial for their reluctance to encourage innovations in the healing art, are beginning to discover the importance of our co-operation in mitigating the woes, and protracting the duration of human existence. The achievement of this object is to be ascribed to the noble spirit of virtuous enterprise with which the present age is inspired, and which we see displayed in every department of human industry and skill. If this spirit is sometimes developed in excess, as when the splendid steam-vessel, ploughing in majesty the billows of the ocean, is followed by the stupid steam-doctor, digging the graves of his victims on the land, it is nevertheless, the consecrated agency by which the destinies of the world are to be gloriously accomplished, and which already has so far transformed the aspect of society, that, could the man who has slept for the last half century, be awakened from his slumbers, he would hardly recognize the world in which we live, as the place of his original abode.

These magnificent results, to appreciate which the man of the present day must traverse oceans by the energies of fire, and fly over plains and mountains on the wings of the wind, are the products of human industry, goaded by a restless enterprize, and guided by resistless genius.

Whether this industry, enterprize and genius, are peculiar to the present age, or whether the existing condition of the Sciences and Arts is the ultimate result of a series of predisposing causes, which have been operating for ages past, is matter of little moment. It is quite sufficient for our happiness and mutual congratulation, to know that we live in an age of unparallelled improvement in all that can result from the energies of intellect acting upon material nature, or from the re-action of matter upon the human mind.

In this condition of civilized man, and of all those Arts and Sciences which embellish life, imparting to it augmented zest and increased duration, it is our happiness to know that neither the theory, nor the practice of the Dental Art, has been left behind the age, either by the incompetency or inactivity of those individuals to whom, in the progress of events, its destiny has been intrusted. If there be any class of men in these States who have "compassed sea and land," in pursuit of usefulness, fame and fortune, braving obloquy, and resisting opposition, enduring fatigue, and despising danger, there are surely many among our professional collaborators who stand in the foremost rank of these benevolent adventurers. Nor have they exerted themselves in vain. Ample fortune and enviable celebrity, have already rewarded the spirited zeal of many labourers in this wide field of professional enterprise; while the improved condition of our Art, as regards its salutary influence on society at large, is a proof of the justice of the desert which has been thus munificently rewarded.

What encouragement, then, is this, to increased exertion in this field of useful labour, and honorable distinction! Who would not prefer to attend these distinguished pioneers in the paths of Dental Science, and share their rewards, rather than follow the Cæsars and Napoleons of past ages to their fields of blood, and their untimely graves?

Presuming that many of those individuals, to whose professional interests this work will be devoted, must feel as we do on the subject of elevating the Dental Science to its deserved position among the callings of men,—and feeling confident that where much has been already done, still more remains yet to be effected, the Publishing Committee have issued this specimen number of a Monthly Periodical, dedicated to the profession at large, with a perfect conviction of the obvious truth, that by their prompt encouragement or silent neglect, it must either stand or fall. That any other class of our fellow citizens, excepting, perhaps, an inconsiderable portion of the medical profession, will afford the least encouragement to the present undertaking, is beyond our hope. The details of Dental Practice, and even the more sufferable theory of our Art, possess an absorbing interest chiefly with the more intelligent and ambitious of our own profession; and it is to them therefore, that we now appeal, by first presenting an expose of our general plan, and then suggesting a few brief considerations to induce our professional brethren to co-operate in our design.

